# Case Report: Interpectoral lymph node metastasis as an indicator of occult breast cancer: a case of double primary malignancy

**DOI:** 10.3389/fonc.2026.1776643

**Published:** 2026-04-15

**Authors:** Qian Zhao, Yangxuzhu Liu, Yifan Fu, Yuheng Wu, Wei Zhang

**Affiliations:** 1 Department of Breast Surgery, General Surgery Center, The First Hospital of Jilin University, Changchun, Jilin, China; 2 The Affiliated Hospital to Changchun University of Chinese Medicine, Changchun, Jilin, China; 3 Department of Cosmetology Plastic Surgery, The First Hospital of Jilin University, Changchun, Jilin, China

**Keywords:** case report, metachronous brain metastasis, multidisciplinary management, multiple primary cancers, neoadjuvant chemotherapy, occult breast cancer, Rotter’s lymph node metastasis

## Abstract

Occult breast cancer (OBC) is a rare clinical entity, characterized by the presence of metastatic adenocarcinoma in the axillary lymph nodes without an identifiable primary tumor in the breast. We present an unprecedented case of synchronous OBC and primary lung cancer, which initially manifested as an isolated interpectoral (Rotter’s) lymph node metastasis. The patient underwent neoadjuvant chemotherapy, followed by wide local excision with sentinel lymph node biopsy, as well as a subsequent wedge resection of the lung primary. The patient declined further recommended adjuvant radiation therapy and genetic testing. Twelve months later, a solitary brain metastasis developed. Histopathological examination confirmed its breast origin, and a craniotomy achieved complete resection. At over 56 months of follow-up, the patient remains recurrence-free. Metastasis to Rotter’s lymph node can be the sole inaugural presentation of OBC. Pathological distinction between synchronous primaries is paramount for accurate diagnosis and management. Importantly, multidisciplinary management must address metachronous metastases in the context of treatment non-adherence.

## 1 Introduction

Breast cancer was the most frequent cancer in women, and it is the leading cause of cancer deaths globally (1). Occult breast cancer (OBC) is an uncommon type of breast cancer, accounting for approximately 0.3% to 1.0% of all breast cancer (2, 3). Unlike other types of breast cancer, patients with OBC typically present with axillary lymphadenopathy in the absence of an identifiable breast mass. For these patients, diagnosis and treatment were often delayed due to atypical presentations, leading to adverse outcomes. The precise pathogenesis of OBC remains incompletely understood. Hypothesized mechanisms include immune-mediated spontaneous regression of the primary tumor, malignant transformation of ectopic breast tissue within axillary lymph nodes, or the failure of contemporary imaging modalities to detect microscopic primary lesions (4–6). Regarding clinical management, treatment strategies largely favor breast-conserving surgery combined with radiotherapy, or axillary lymph node dissection followed by rigorous surveillance. These conservative approaches yield oncologic outcomes comparable to those of traditional mastectomy (3). Additionally, the selection of systemic therapy is dictated by the immunohistochemical (IHC) receptor status of the metastatic lymph nodes.

Although OBC presenting as enlarged axillary lymph nodes is occasionally seen, the presentation as an enlarged interpectoral (Rotter’s) lymph node is a rare finding. To our knowledge, this is the first case of OBC with Rotter’s lymph node metastasis. Furthermore, the complexity of this case is compounded by the presence of synchronous primary lung cancer (PLC) and subsequent solitary brain metastasis from breast cancer (BCBM).

Multiple primary cancers (MPCs) are the independent development of two or more malignant tumors in different organs (7). With the improved survival rates of cancer patients and advancements in tumor screening technologies, the incidence of MPCs is becoming increasingly common. The diagnosis of MPCs poses challenges, as accurately distinguishing between metastatic disease and a second primary malignancy is critical for formulating appropriate treatment strategies and evaluating prognosis.

The most frequent sites of breast cancer metastasis are the bone, lung, liver and brain. BCBM occurs in approximately 10–30% of patients with metastatic breast cancer (8). Based on anatomical location, BCBM can be classified as choroid plexus metastasis, leptomeningeal metastasis, or parenchymal metastasis, the latter being most common. Based on lesion number, BCBM can be further categorized as solitary or multiple metastases, with over 78% of cases presenting as multiple parenchymal lesions (9). Brain metastasis is commonly associated with poor survival and prognosis. However, this case achieved long-term survival without radiotherapy—highlighting an unusual and favorable outcome.

We formulated an individualized therapeutic approach through multidisciplinary collaboration. The patient achieved satisfactory clinical outcomes and revealed superior treatment tolerance, with no serious adverse effects observed. We describe a case to illustrate the importance of considering OBC when interpreting Rotter’s lymph node metastasis.

## 2 Case presentation

### 2.1 Initial diagnosis and treatment

**Figure 1:**
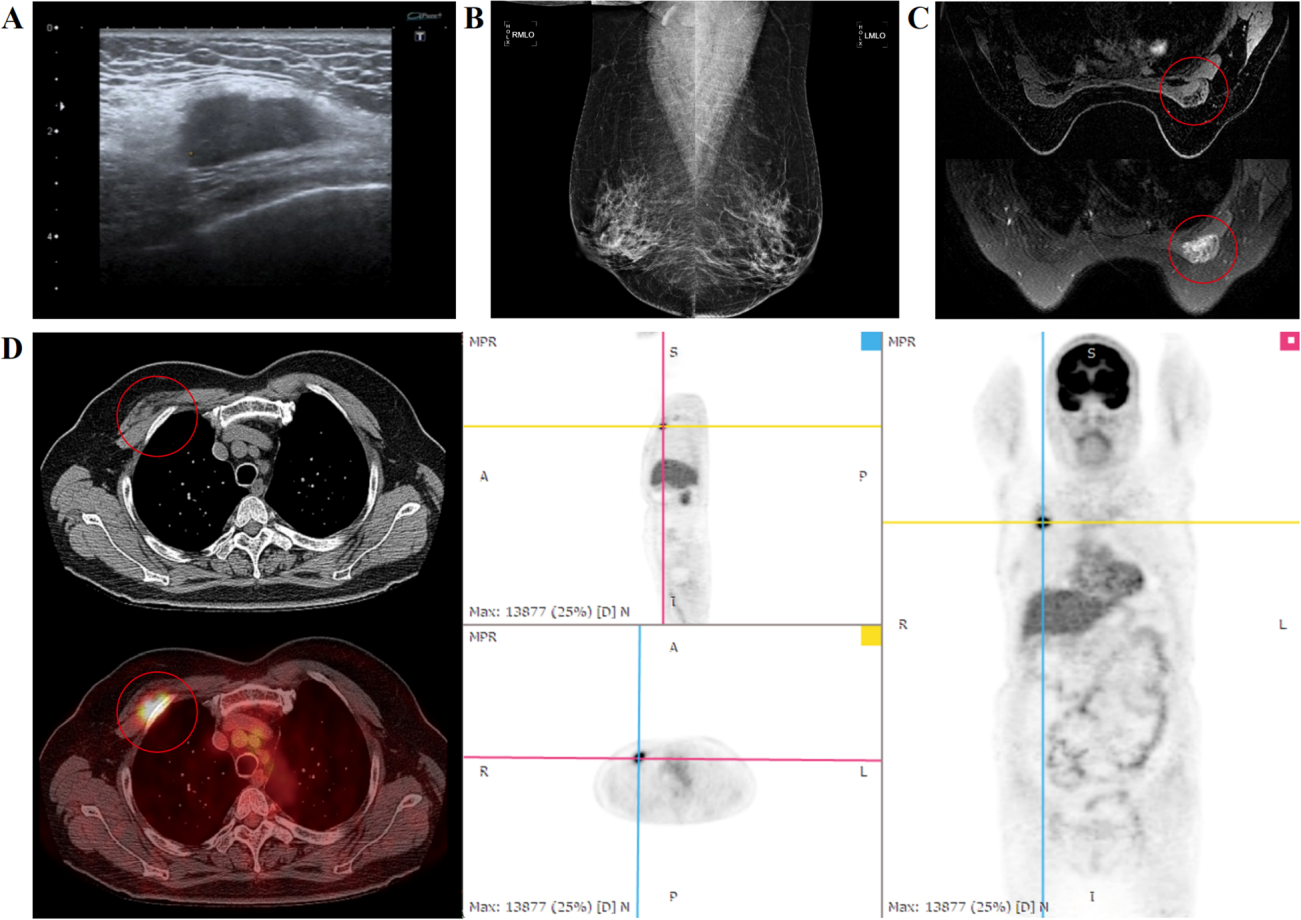
Occult Breast Cancer accompanied by interpectoral (Rotter’s) lymph node. **(A)** Ultrasound showing an enlarged interpectoral lymph node. **(B)** Mammography showing an enlarged interpectoral lymph node. **(C)** MRI showing enlarged interpectoral lymph node. **(D)** PET/CT showing an enlarged interpectoral lymph node, radioactively concentrated (circle), as well as no evident metabolically active lesion in the bilateral breasts.

In April 2019, a 59-year-old female patient presented with an enlarged lymph node incidentally discovered in the right chest wall. Core-needle biopsy of the interpectoral lymph node was performed, and the pathological outcome revealed carcinoma in connective tissue. IHC indicated: ER (-), PR (weakly positive, 30%), Her-2 (4B5) (2+) with negative gene amplification, Ki-67 (+30%), GATA-3 (+), GCDFP-15 (+), CK7 (+), CA15-3 (+), TTF-1 (-), CK20 (-), and Villin (-). The IHC profile strongly supports a breast origin. GATA-3 shows high sensitivity for breast carcinoma, while GCDFP-15 provides additional specificity (10). The CK7-positive/CK20-negative pattern further favors a primary breast tumor over gastrointestinal sources, while negative TTF-1 and Villin help exclude lung and intestinal origins. Considering the metastatic nature of the Rotter’s lymph node, a comprehensive breast evaluation was performed to detect the primary lesion, including breast ultrasound (**Figure 1A**), mammography (**Figure 1B**), and magnetic resonance imaging (MRI) (**Figure 1C**). Notably, all imaging modalities failed to detect any primary breast lesion. Subsequent 18F-deoxyglucose positron emission tomography/computed tomography (18F-FDG PET/CT) (**Figure 1D**) revealed: 1) enlarged lymph node in the intermuscular space of the right pectoralis major and minor muscles considered malignant, measuring approximately 1.5cm x 2.5cm, with CT value of about 29 HU and increased FDG metabolism (standardized uptake value (SUV) max = 9.2); 2) a ground-glass nodule in the right upper lung, measuring approximately 0.9 cm x 1.2 cm, with CT value of approximately -524 HU and no abnormal FDG metabolism, but malignancy could not be excluded. After thorough assessment, the patient was diagnosed with OBC.

Upon consultation with a multidisciplinary team (MDT) encompassing experts in breast surgery, thoracic surgery, medical oncology, radiology, and pathology, the right upper lobe ground-glass nodule was deemed highly suspicious for PLC, pending histopathological verification. The MDT devised a sequential therapeutic approach. The initial phase prioritized neoadjuvant chemotherapy (NAC) and surgical intervention for the OBC, deferring the management of the pulmonary lesion to a subsequent phase. Tailored to the patient’s immunohistochemical profile, an 8-cycle AC-T neoadjuvant regimen utilizing liposomal doxorubicin and cyclophosphamide followed by albumin-bound paclitaxel was implemented.

**Figure 2:**
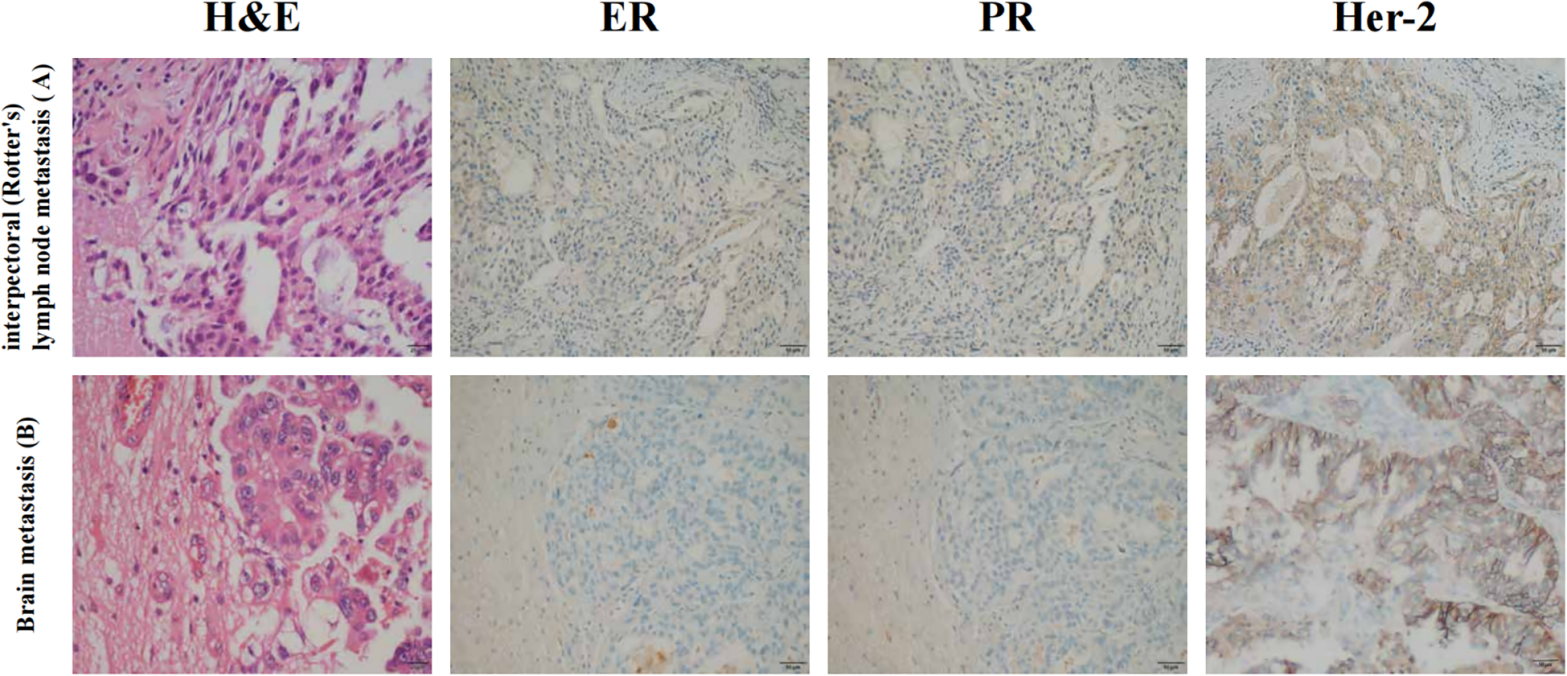
Histopathological and immunohistochemical images from the **(A)** interpectoral lymph (Rotter’s) node metastasis and **(B)** brain metastasis.

Treatment efficacy was monitored serially using ultrasound to assess disease progression. The patient completed 8 cycles of NAC with the AC-T regimen. Imaging evaluation showed a significant reduction in lesion size. According to Response Evaluation Criteria in Solid Tumors (RECIST 1.1), the treatment response was categorized as partial response (PR). Following repeat MDT discussion, which reviewed treatment response, the decision was made to proceed with wide local excision for the metastatic carcinoma in the interpectoral lymph node along with sentinel lymph node biopsy. The final pathology report revealed minimal residual invasive carcinoma. All surgical margins were negative, and no metastatic carcinoma was identified in the sentinel lymph nodes (0/5). The minimal size of the residual lesion post-neoadjuvant chemotherapy, along with crush artifacts, precluded detailed evaluation of tumor morphology, growth pattern, and lymphovascular invasion. IHC (**Figure 2A**) results were as follows: Ki-67 (+40%), ER (-), PR (-), Her-2 (4B5) (2+), which were inconsistent with the biopsy findings.

During treatment for OBC, the patient underwent lung computed tomography (CT) scans every two to three months to monitor changes in the pulmonary lesion. Serial imaging revealed progressive changes in the right upper lobe lesion, including a slight increase in size and focal areas of increased density. The MDT reconvened to review the evolving imaging findings and recommended thoracoscopic wedge resection with lymph node dissection for definitive diagnosis and management. Postoperative pathology confirmed the diagnosis of primary microinvasive lung adenocarcinoma (measuring 0.9 cm × 0.8 cm × 0.8 cm), with no evidence of metastatic involvement in any of the excised lymph nodes (groups 2, 4, 7, 10, and 11). IHC showed a typical lung adenocarcinoma profile: CK7 (+), TTF-1 (+), Napsin A (+), CK20 (-), and a low Ki-67 proliferation index (10%). TTF-1 and Napsin A both had high positive predictive value and diagnostic accuracy for adenocarcinoma of the lung. Importantly, the specificity of Napsin A for adenocarcinoma was higher than that of TTF-1 (11). Given the genetic predisposition underlying MPCs—such as mutations in genes like BRCA1/2, TP53, and EGFR, we recommended genetic testing to inform subsequent targeted therapies, immunotherapies, and risk assessment. However, the patient declined genetic testing due to personal and financial reasons. Postoperatively, the patient continued to have regular follow-up, including pulmonary CT scans and tumor marker assessments. To date, there has been no radiologic or laboratory evidence to indicate local recurrence or distant metastasis of the lung cancer.

### 2.2 Brain metastasis diagnosis and treatment

**Figure 3:**
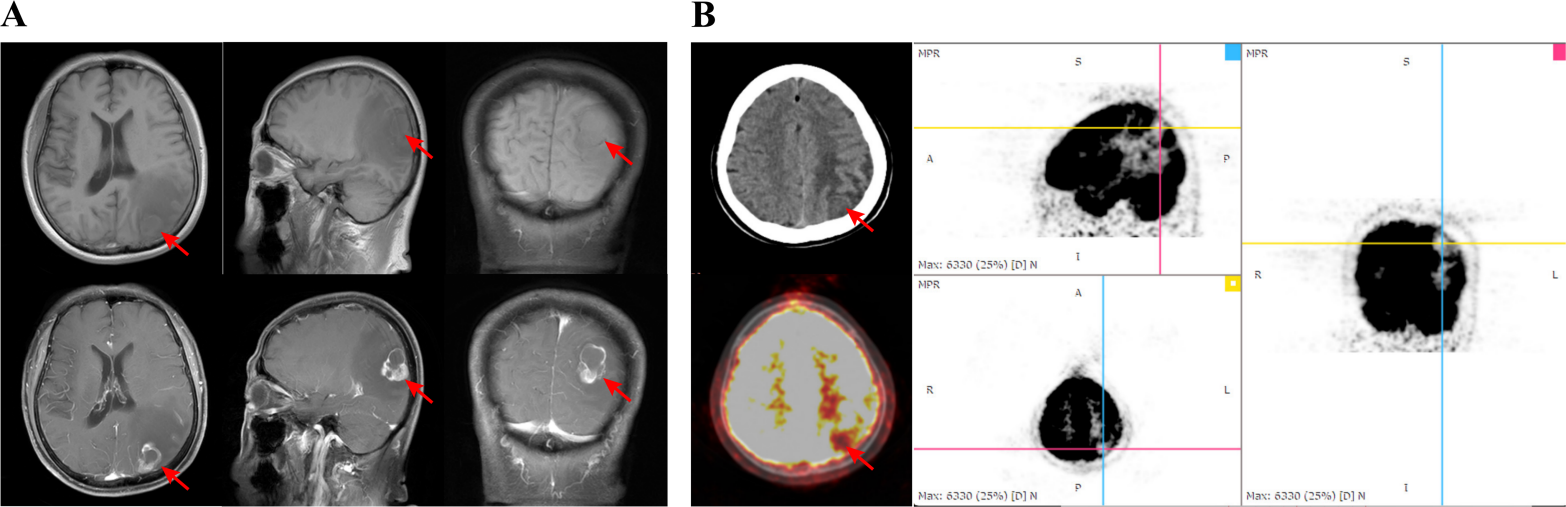
Solitary brain metastasis. **(A)** Non-contrast and contrast-enhanced T1-weighted MRI in the axial, coronal and sagittal planes showed a focal solid tumor in the left parieto-occipital lobe (arrow). **(B)** PET/CT showing evidently reduced radiosensitivity centrally and increased uptake in the peripheral ring (arrow).

In October 2020, the patient presented with symptoms of head swelling and pain, and no associated nausea, vomiting, or altered consciousness. Cranial MRI (**Figure 3A**) revealed an abnormal signal in the left parieto-occipital lobe. An ^18^F-FDG PET/CT scan (**Figure 3B**) showed a metabolically active, cystic-solid nodule in the left parieto-occipital lobe measuring approximately 2.4 cm × 1.4 cm. The lesion showed reduced radiosensitivity centrally and increased uptake in the peripheral ring (SUVmax = 10.8; CT value = 36.3 HU), consistent with features of a metastatic brain tumor. Neurological examination revealed normal muscle strength and tone, intact physiological reflexes, with no pathological reflexes.

The patient subsequently underwent resection of the cerebral lesion with dural patch repair in the neurosurgery department. Postoperative histopathology confirmed the diagnosis of BCBM. IHC (**Figure 2B**) revealed GATA-3(+), Ki-67 (+60%), CK20 (-), CK7 (+), ER (-), Her-2 (4B5) (2+), PAX-8 (-), PR (-), TTF-1 (-), and Villin (-), consistent with an adenocarcinoma derived from breast tissue.

### 2.3 Follow-up

**Figure 4:**
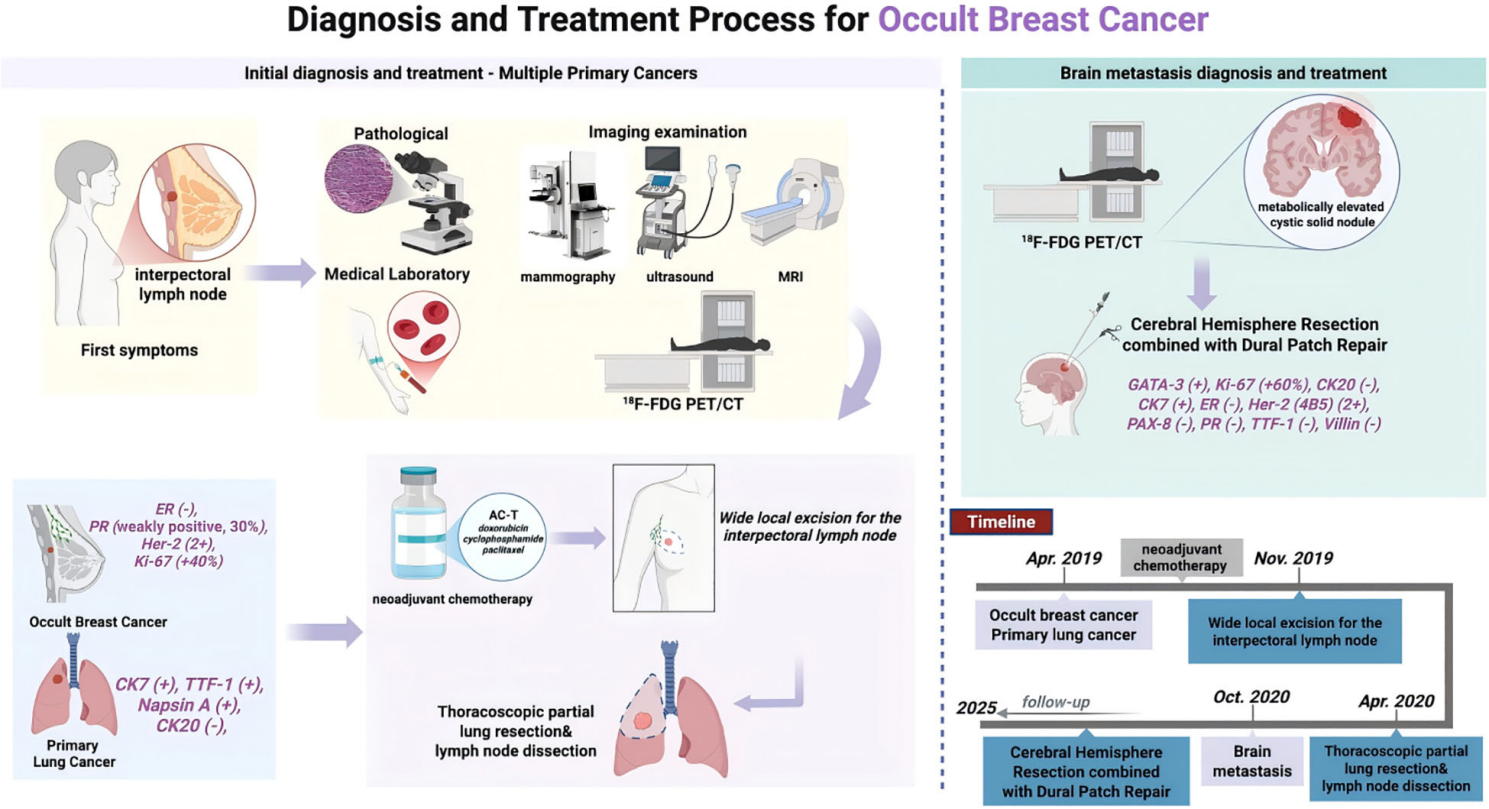
Diagnosis and treatment process of a case of occult breast cancer: Illustrated are the diagnostic steps (including medical laboratory, imaging examination, and pathological), neoadjuvant chemotherapy, surgical interventions, the management of a synchronous primary lung cancer, and the subsequent development of brain metastasis. Created in BioRender. (Created in BioRender. https://BioRender.com/f7dsj71).

As of September 2025, the patient has survived more than 56 months since the diagnosis of BCBM. During follow-up, imaging studies—including ultrasound, mammography, and MRI—as well as tumor marker assessments, revealed no significant abnormalities. The patient exhibited no cognitive or motor deficits, no symptoms of additional neurological impairment, and no evidence of local tumor recurrence or distant metastasis. The patient’s diagnostic and therapeutic course is summarized in (**Figure 4**).

## 3 Discussion

OBC is a rare subtype of breast cancer, characterized by the absence of an identifiable primary tumor within the breast on imaging examination. This unique entity was first described by Halsted in 1907 (12). Nonetheless, available clinical data are limited because of its low incidence, which has resulted in a lack of large-scale studies. Consequently, significant challenges remain in the areas of early diagnosis, accurate staging, and developing individualized treatment strategies for OBC.

The clinical manifestations of OBC are nonspecific, with the most common initial presentation of which is painless axillary lymphadenopathy. In some cases, OBC presents as lymph node metastasis in specific locations. Based on a systematic search of the PubMed and Web of Science databases, we reviewed relevant literature published in the past decade. **Table 1** summarizes the clinical characteristics and treatment approaches of occult breast cancer presenting with lymph node metastases in specific locations.

**Table 1:** Literature review of occult breast cancer with lymph node metastases in specific locations.

Publication year	Age	Sex	Clinical symptom	Management	Prognosis	Reference
2024	45	Female	retro-scapular area lymph node metastasis	Chemotherapy Targeted therapy Surgery Hormone therapy Radiotherapy	Good outcome	(13)
2024	62	Female	right axillary and supraclaicular lymph nodes metastasis	Chemotherapy Targeted therapy Surgery Hormone therapy Radiotherapy	Good outcome	(14)
2023	63	Female	left axillary and right inguinal lymph nodes	Not mentioned	Not mentioned	(15)
2022	54	Female	retroperitoneal lymph node lymph nodes metastasis	Not mentioned	Not mentioned	(16)
2019	68	Female	mediastinal and abdominal lymph node metastasis	Chemotherapy Targeted therapy	Good outcome	(17)
2017	67	Male	right axilla and neck lymph node metastasis	Chemotherapy Targeted therapy	Good outcome	(18)
2017	33	Female	intramammary lymph node metastasis	Chemotherapy Surgery Radiotherapy	Good outcome	(19)
2016	50	Female	left collarbone and axilla lymph nodes metastasis	Chemotherapy Targeted therapy	Good outcome	(20)

### 3.1 Unusual initial presentation: the significance of Rotter’s lymph node metastasis

This case is notable for its initial presentation of isolated interpectoral (Rotter’s) lymph node metastasis. These lymph nodes, located within the interpectoral fascia between the pectoralis major and minor muscles, were first described by Grossman in 1896 and later documented by Rotter in 1899 (21). While studies have shown a higher incidence of Rotter’s lymph node metastasis in patients with positive axillary lymph nodes (22), this case is unique due to the isolated involvement of these nodes as the first sign of OBC. To our knowledge, this is the first reported case of OBC presenting in this manner.

Although the American Joint Committee on Cancer (AJCC) staging system classifies Rotter’s nodes as a subsegment of Level II axillary lymph nodes, their clinical significance is often under-recognized. Their deep anatomical location makes them impalpable during routine physical examinations unless they are significantly enlarged. Furthermore, standard breast imaging, such as mammography and ultrasound, typically focuses on the mammary parenchyma and axillary tail, often overlooking the interpectoral region. This contributes to a low index of suspicion, leading to potential misdiagnosis of a mass in this area as a benign soft-tissue tumor. This case underscores the crucial diagnostic principle: metastasis to Rotter’s lymph nodes must be considered in the differential diagnosis for any mass in the interpectoral region.

### 3.2 OBC: navigating therapeutic management complexities

In accordance with the current National Comprehensive Cancer Network (NCCN) Clinical Practice Guidelines for Breast Cancer (v4, 2025), treatment decisions for OBC patients with negative breast MRI findings should be stratified by lymph node status, referencing the management of corresponding invasive breast cancer. As existing evidence suggests, optimizing locoregional management in OBC requires careful consideration of NAC, RT, and the surgical strategy.

Despite the occult nature of the primary tumor, NAC is a cornerstone of OBC management. It reduces tumor burden, downstages lesions for surgery, and allows for the assessment of chemosensitivity through serial monitoring of metastatic sites. Recent studies suggest that NAC is a reasonable option for OBC patients, particularly those with triple-negative disease, which has a higher likelihood of achieving a pathological complete response (pCR) (3).

The comparative efficacy of breast-conserving surgery (BCS) versus mastectomy remains debated regarding to surgical approach to the breast. While one study reported no significant survival difference (p=0.70) (23), another indicated that superior disease-free survival (DFS) with mastectomy combined with axillary lymph node dissection (ALND) compared to BCS combined with ALND (24). Contemporary management trends reflect a modest shift toward less aggressive axillary surgery, with SLNB use increasing from 4.7% in 2012 to 16.2% in 2021 among OBC patients (25). A retrospective study of cT0N+ OBC patients indicated that ALND may confer a survival benefit over sentinel lymph node biopsy (SLNB) in patients who did not receive NAC (overall survival (OS) 106.9 vs 85.5 months, p = 0.013) (26). However, this advantage seems to be attenuated in patients who receive NAC. In addition, a recent analysis of the National Cancer Database examining 2,759 OBC patients demonstrated that among those achieving nodal pCR after NAC, there was no significant difference in overall survival (OS) between ALND, SLNB + ALND, and SLNB alone groups. There was no difference in OS with respect to axillary surgical procedure in those with nPCR after NAC. This suggests that for carefully selected OBC patients with an excellent clinical response to NAC and negative SLNB, omission of ALND may be considered (25). Furthermore, RT reveals a substantial survival benefit following SLNB in NAC-treated patients (123 months with RT vs 64 months without RT, p = 0.034) (26). Consequently, for patients with OBC who did not undergo mastectomy, postoperative whole-breast irradiation (WBI) is recommended. Collectively, these findings underscore that optimal treatment decisions for OBC must integrate NAC response, planned RT, and patient-specific factors, such as preference for breast conservation and tumor biology.

### 3.3 Brain metastasis: risk assessment and multimodal management

In accordance with epidemiological data, breast cancer generally ranks the second most common tumor of brain metastatic origin after lung cancer (27). For clinicians, it’s critical to accurately differentiate non-neoplastic lesions, primary brain lesions, brain metastases, and the origin of the metastatic tumor to guide therapeutic decisions. Managing BCBM is challenging due to tumor heterogeneity, circulating tumor cells (CTCs), and the unique barriers posed by the blood-brain barrier (BBB) and blood-tumor barrier (BTB) (28). Current multimodal approaches, encompassing surgical resection, stereotactic radiosurgery (SRS), whole-brain radiation therapy (WBRT), systemic therapy, and targeted agents, require personalized selection (29). The advances in systemic therapy have expanded treatment options for BCBM. A multi-institutional study of 26 patients with HR+/HER2- and triple negative BCBM treated with sacituzumab govitecan (SG) concurrent with SRS indicated excellent local control rates of 94% at 12 months and 84% at 24 months, with no cases of symptomatic radiation necrosis. The median central nervous system progression-free survival was 5.4 months, and median overall survival was 8.4 months, suggesting that combining antibody-drug conjugates with SRS may offer a safe and effective strategy for BCBM management (30). In this case, surgical resection was prioritized due to isolated brain parenchymal lesions and the patient’s refusal of radiation. It’s crucial to recognize that central nervous system (CNS) invasion signifies advanced disease and high tumor aggressiveness. This phenomenon further underlines the necessity for rigorous post-BCBM surveillance to detect extracranial metastases (e.g., liver, lung, bone) and local recurrence.

### 3.4 MPCs: from diagnostic difficulty to genetic risk assessment

The patient’s diagnosis of synchronous primary lung and breast cancers represents a case of MPCs. The rarity of MPCs, combined with the absence of sensitive and specific markers, frequently brings about missed or incorrect diagnoses. Standardized diagnostic criteria remain undefined, though most researchers adopt a consensus that requires each tumor to have a distinct malignant pathological diagnosis, be anatomically separate, and show no evidence of metastasis between them. Nowadays, the majority of domestic and international researchers adopt the diagnostic criteria which include the following criteria: 1) Each tumor must have a malignant pathological diagnosis; 2) Each tumor must originate from different tissues or organs, exist independently, and be separated by normal tissue; and 3) There must be confirmation that there is no metastasis between the tumors (31).

Breast cancer and lung cancer are the two most common cancers characterized by the most conspicuous morbidity and mortality for women, and there is a correlation between them. Previous studies (32) have demonstrated that approximately 1% of female patients with primary breast cancer later developed PLC. Factors such as ER-/PR- breast cancer, short latency (within 1 year after breast cancer diagnosis), younger age at breast cancer diagnosis, Black individuals, poorly or undifferentiated histological grade of breast cancer immensely heighten the risk of developing PLC in these patients. As these findings demonstrate, there may be common risk factors for the development of breast cancer and lung cancer. From a more in-depth standpoint of genetic mechanisms, other than common susceptibility genes such as BRCA2, TP53 and RAD51D, rare cancer-related variations like EXT2, WWOX, GATA2, and GPC3 are also found in patients with dual primary cancers of breast cancer and lung cancer, thereby reinforcing the core role of genetic factors in the formation of MPCs (33). According to a genetic association study, individuals carrying a rare pathogenic variant in one of 16 cancer-associated genes were 1.9 times more likely to develop a single cancer and 2.6 times more likely to develop MPC (34). Therefore, for patients with confirmed MPCs, genetic testing is recommended to provide genetic counseling and individualized risk assessment.

### 3.5 Outcome analysis: factors influencing clinical course

The patient’s decision to decline genetic testing and adjuvant radiation therapy (RT) due to personal and financial constraints limited the implementation of stratified disease management and accurate prognostic assessment. Despite these limitations, the patient had a favorable response to therapy, resulting in sustained disease stabilization. This positive outcome can be attributed to several key factors.

First, complete surgical resection with pathologically negative margins was achieved. Additionally, the tumor microenvironment (TME) displayed less favorable immunosuppressive activity, characterized by diminished expression of immune checkpoint molecules (e.g., PD-L1), abundant tumor-infiltrating lymphocytes (TILs) in a non-exhausted functional state, and attenuated pro-tumorigenic functions of regulatory T cells (Tregs) and M2-polarized tumor-associated macrophages/microglia, collectively fostering robust anti-tumor immunity. In addition, surgical debulking considerably lessened tumor burden and eliminated a primary source of immunosuppressive factors, which created a TME advantageous for the clearance of residual micrometastases by endogenous immune surveillance. Additional contributing factors included the patient’s young age, good performance status, absence of significant comorbidities, and exceptionally close postoperative monitoring. The combined effect of surgical cytoreduction and a permissive immune microenvironment may have compensated for the absence of RT, thereby achieving effective local control and a favorable prognosis (35, 36). Nonetheless, it is crucial to underline that this scenario represents a highly selected case and cannot be generalized. Postoperative RT remains the standard of care for patients suffering from brain metastases to minimize recurrence risk. For carefully selected patients who forego RT, long-term and rigorous follow-up is essential.

## 4 Conclusion

Breast cancer metastasis should be suspected in patients presenting with an enlarged Rotter’s lymph node. More importantly, sequential treatment guided by dynamic disease monitoring is recommended for patients presenting with two or more primary malignant tumors, prioritizing highly aggressive or life-threatening lesions. In cases of isolated parenchymal brain metastases from OBC, surgical resection, particularly when complete (R0) resection is achievable, may be a viable strategy to improve overall survival.

## Data Availability

The original contributions presented in the study are included in the article/supplementary material. Further inquiries can be directed to the corresponding author.
